# Role of JNK and ERK1/2 MAPK signaling pathway in testicular injury of rats induced by di-*N*-butyl-phthalate (DBP)

**DOI:** 10.1186/s40659-019-0248-1

**Published:** 2019-08-06

**Authors:** Hongyan Wang, Weipeng Zhou, Jing Zhang, Huan Li

**Affiliations:** 1 School of Public Health, Beihua University, Jilin, 132013 China; 2 The First Clinical Medical College of Nanchang University, Nanchang, 330006 China; 3 Department of Environmental Hygiene, School of Public Health, Beihua University, Jilin, 132013 China

**Keywords:** Di-*N*-butyl-phthalate, Testis, Sertoli cells, Mitogen-activated protein kinase

## Abstract

**Background:**

Di-*N*-butyl-phthalate (DBP) is an endocrine disrupting substance. We investigated the adverse effect of DBP on testis of male rat and reveal its potential mechanism of MAPK signaling pathway involved this effect in vivo and in vitro. Gonadal hormone, sperm quality, morphological change and the activation status of JNK, ERK1/2 and p38 was determined in vivo. Primary Sertoli cell was established and cultivated with JNK, ERK1/2 inhibitors, then determine the cell viability, apoptosis and the expression of p-JNK, p-ERK1/2. Data in this study were presented as mean ± SD and determined by one-way analysis of variance (ANOVA) followed by Bonferroni’s test. Difference was considered statistically significant at *P *< 0.05.

**Results:**

In vivo experiment, DBP impaired the normal structure of testicular tissue, reduced testosterone levels in blood serum, decreased sperm count and increased sperm abnormality, p-ERK1/2 and p-JNK in rat testicular tissue increased in a dose-dependent manner. In vitro studies, DBP could decrease the viability of Sertoli cells and increase p-ERK1/2 and p-JNK. Cell apoptosis in SP600125 + DBP group was significantly lower than in DBP group (P < 0.05). p-JNK was not significantly decreased in SP600125 + DBP group, while p-ERK1/2 was significantly decreased in U0126 + DBP group.

**Conclusions:**

These results suggest that DBP can lead to testicular damage and the activation of ERK1/2 and JNK pathways, the JNK signaling pathway may be primarily associated with its effect.

## Background

Mounting evidence has implicated that Phthalic acid esters (PAEs), as a widely used synthetic compound, may be related to carcinogenesis, inflammation, metabolic disorder and especially reproductive and developmental disease [1]. Dibutyl phthalate (DBP), one of the most used PAEs, has been proved to be an endocrine disrupting chemical (EDC) which displays estrogenic or anti-androgenic activity in male reproductive system, leading to testicular atrophy, seminiferous tubule degeneration, germ cell loss and infertility [2]. In 1983, animal experiments conducted by Oishi S have shown that di-2-ethylhexyl phthalate (DEHP) can cause testicular atrophy in young male rats [3]. In vivo, DBP acts by reducing cell proliferation and impairing differentiation through reducing expression levels of Pou5f1 and Mki67 in prepubertal and pubertal testes [4]. In compare to other EDCs including other Phthalates and the mixture of EDCs, the main effect of DBP was decrease intratesticular testosterone, and steroid hormone enzymes [5]. It can be seen from the above research results that the association of DBP exposure with male fertility problems is the concerning problems of this and others toxicologicals research in this field.

Testis injury induced by DBP is characterized with testis atrophy and seminiferous epithelium degeneration [6]. It was reported that DBP could penetrate into testis despite the existence of blood-testis barrier (BTB) which is one of the tightest physiological barriers in mammals [7]. Sertoli cells which form the BTB provide the germ cells with nutrition, structure support and protective shield [8]. Studies showed the distribution of vimentin cytoskeleton and intercellular junction proteins in Sertoli cells were altered by DBP, inducing spermatogenic cells to detach from Sertoli cells and then undergo apoptosis [9]. These studies may indicate that Sertoli cell could be the primary target of DBP in testis. However, the properties of Sertoli cells are modulated by various substances such as steroid hormone, cytokine, and protein kinase. Therefore elucidating the mechanisms underlying the impair effect of DBP to Sertoli cells would give hints to the prevention of EDCs induced male reproductive dysfunction.

MAPK signaling pathway is one of the important signal transduction systems, involved in cell proliferation, differentiation, apoptosis and response to environmental stimuli [10]. In mammalian testis MAPK can regulate cell proliferation, differentiation and apoptosis, is considered one of the important determinants of sperm development. MAPK can indirectly affect the development of animal germ cells by influencing the function of Sertoli cells [11]. There are three MAPK units in the mammalian cells, namely, c-Jun N-terminal kinase (JNK), p38, extracellular signal regulated kinase (ERK) subfamily [12]. Sertoli cell proliferation and differentiation are mainly regulated by follicle stimulating hormone (FSH), whereas FSH activity is inseparable from ERK signaling cascade [13]. Cells respond to many reproductive toxicants by activating the MAPK pathway, for example, bisphenol A induces apoptosis by activating ERK and JNK signaling pathways [14], 1–3-dinitrobenzene can induce apoptosis in TM4 mouse Sertoli cells by JNK-MAPK pathway [15]. 4-nonylphenol isomers can induce apoptosis of mouse Sertoli TM4 cells by activating mitogen-activated protein kinase pathway [16]. Therefore, we speculate that the effect of DBP on Sertoli cells may also be related to the MAPK signaling pathway.

## Results

### DBP decreases testosterone level in blood serum

As revealed in this study (Table 1), the level of testosterone in blood serum decreased whereas FSH and LH increased in the DBP treated groups. Compared with the solvent control group, the level of serum FSH in middle and high dose DBP group increased, and the level of serum LH in high dose DBP group was significantly higher (P < 0. 05). The serum levels of testosterone in middle and high dose DBP group were significantly lower than those in solvent control group (P < 0.05).

**Table 1:** The level of FSH, LH and T of rat serum in each group

Group	FSH (IU/L)	LH (IU/L)	T (nmol/L)
Control	5.94 ± 1.08	12.54 ± 3.16	11.86 ± 1.82
50 mg/kg DBP	6.27 ± 1.82	12.86 ± 4.13	10.05 ± 1.32
500 mg/kg DBP	10.39 ± 1.46*	14.28 ± 3.66	6.65 ± 0.97*
1000 mg/kg DBP	13.56 ± 2.02*	16.68 ± 4.08*	3.57 ± 0.54*

*P < 0.05 vs. the solvent control group; *n *= 8 in each group

### DBP disrupts spermatogenesis

DBP can reduce the sperm count and sperm viability of rats and increase the Malformation rate (see Table 2). Compared with the solvent control group and the low-dose group, the sperm count and sperm viability of the rats in the high dose DBP group were significantly lower (P < 0.05). The total abnormal rate of spermatozoa in the middle and high dose DBP group was significantly higher than that in the solvent control group (P < 0.05). There were statistically significant on the rat sperm count and total sperm abnormality rate between the medium and high DBP dose group (P < 0.05). The dose response relationship is shown in the sperm malformation rate.

**Table 2:** The count, viability and malformation rates of sperm in each group

Group	Sperm count (10^5^/mL)	Viability rate (%)	Malformation rate (%)
Control	1368.56 ± 130.42	83.63 ± 15.10	4.35 ± 0.93
50 mg/kg DBP	1281.93 ± 126.03	79.84 ± 13.35	6.06 ± 0.78
500 mg/kg DBP	1207.94 ± 112.75	76.80 ± 12.52	13.64 ± 1.68*^#^
1000 mg/kg DBP	631.26 ± 106.49*^#a^	64.71 ± 11.36*^#^	19.54 ± 2.86*^#a^

*P < 0.05 vs. the control group; ^#^*P *< 0.05 vs. ^#^*P *< 0.05 vs. 50 mg/kg DBP groups; ^a^*P *< 0.05 vs. 500 mg/kg DBP groups; *n *= 8 in each group

### DBP induces seminiferous tubules degeneration

**Fig. 1:**
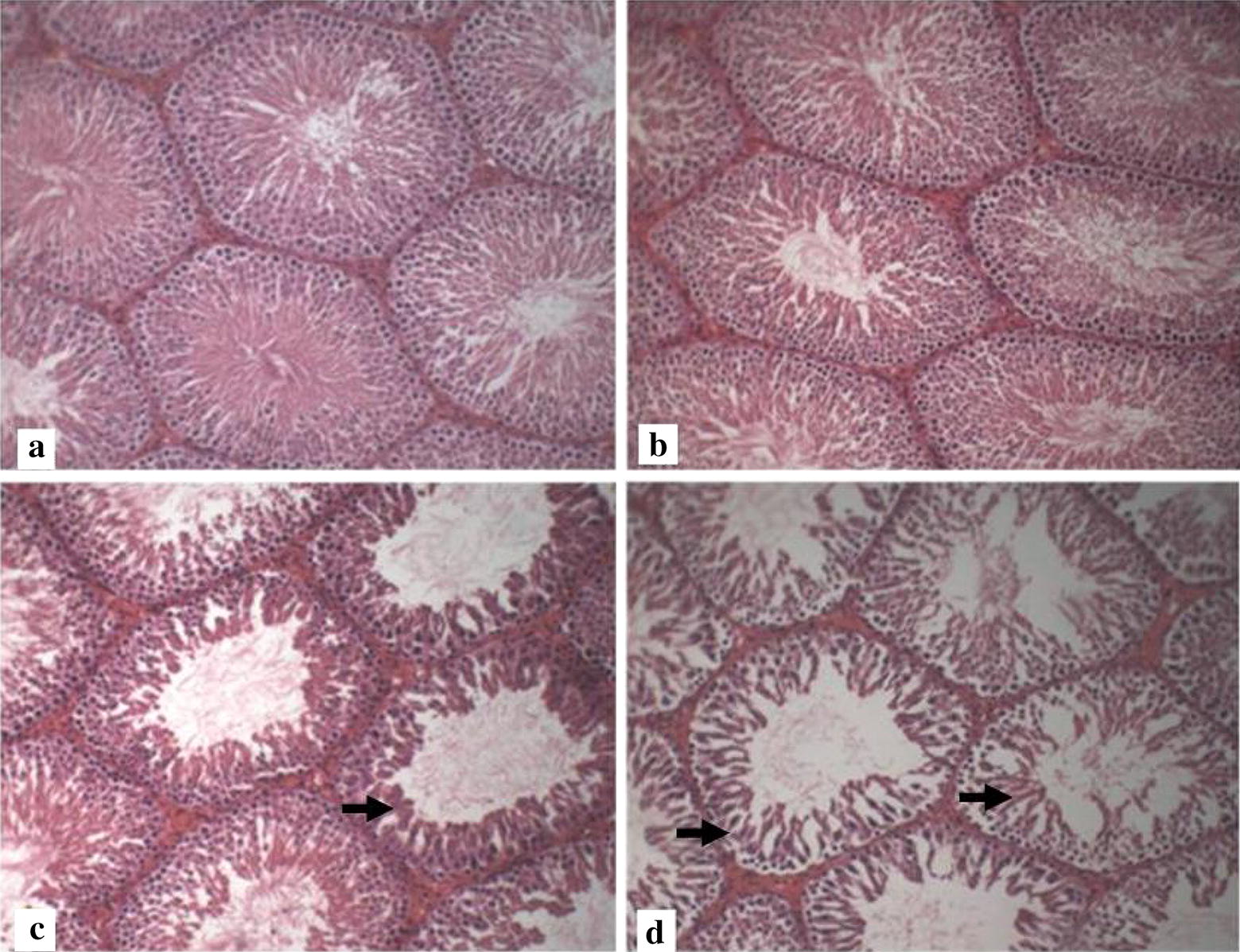
Histomorphological effects of DBP on testis in rats (HE, x 200).** a** The solvent control group, the testicular seminiferous tubules were arranged and the structure was complete. No cells were shed in the lumen, and the number and structure of the sertoli cells were normal. **b** The testis tissue structure of the rats in the low dose DBP group was not changed obviously .** c** The middle dose DBP group, the testicular seminiferous tubules arranged in the rule, but the diameter became smaller, the interstitial widened, the level of spermatogenic epithelium decreased, and the spermatogenic cell shedding phenomenon was seen.** d** High-dose DBP group testicular seminiferous tubules arranged irregularly, spermatogenic epithelial cells severely damaged, sertoli cells dropped, and showed a vacuolar change

In the solvent control group, the testicular seminiferous tubules were arranged and the structure was complete. No cells were shed in the lumen, and the number and structure of the Sertoli cells were normal (Fig. 1a). Compared with the control group, the testis tissue structure of the rats in the low dose DBP group was not changed obviously (Fig. 1b). In the middle dose DBP group, the testicular seminiferous tubules arranged in the rule, but the diameter became smaller, the interstitial widened, the level of spermatogenic epithelium decreased, and the spermatogenic cell shedding phenomenon was seen (Fig. 1c). High-dose DBP group testicular seminiferous tubules arranged irregularly, spermatogenic epithelial cells severely damaged, Sertoli cells dropped,and showed a vacuolar change (Fig. 1d).

### DBP induces activation of JNK and ERK1/2 signaling pathway in the testes

**Fig. 2:**
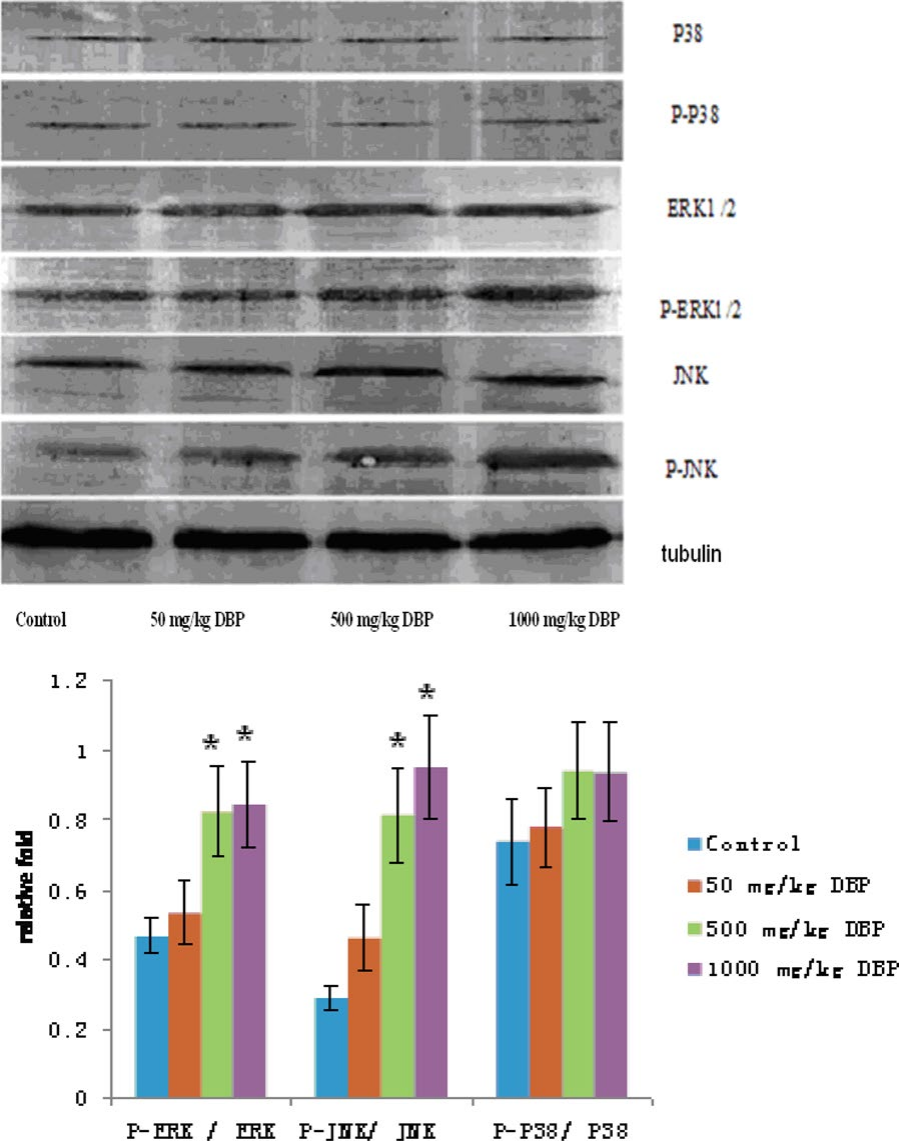
MAPK signaling pathway-related protein expression levels in testis. *P < 0.05 vs. the control group; *n *= 8 in each group

Western blot results showed that DBP can induce the activation of ERK1/2 and JNK in the MAPK signaling pathway in testicular tissue (see Fig. 2). The ratio of the optical density of the phosphorylated protein to the corresponding total protein of the MAPK signaling pathway in the rat testis was seen in Table 3. There was no statistical difference on p-P38/P38 in the testes between DBP groups and the control group. The ratio p-ERK/ERK and p-JNK/JNK in the testes of middle and high dose DBP group was significantly increased than those in control group and low-dose DBP group (P < 0.05) (see Fig. 2).

**Table 3:** The ratio of the optical density of MAPK signaling pathway-related phosphorylated protein to corresponding total protein in Rat testes

Group	P-ERK/ERK	P-JNK/JNK	P-P38/P38
Control	0.4651 ± 0.0521	0.2895 ± 0.0357	0.7368 ± 0.1219
50 mg/kg DBP	0.5319 ± 0.0935	0.4615 ± 0.0956	0.7778 ± 0.1151
500 mg/kg DBP	0.8235 ± 0.1312*^#^	0.8139 ± 0.1342*^#^	0.9412 ± 0.1376
1000 mg/kg DBP	0.8462 ± 0.1225*^#^	0.9512 ± 0.1468*^#^	0.9375 ± 0.1426

*P < 0.05 vs. the control group; ^#^*P *< 0.05 vs. 50 mg/kg DBP group, *n *= 8 in each group

### Effects of DBP on Sertoli cell viability

**Fig. 3:**
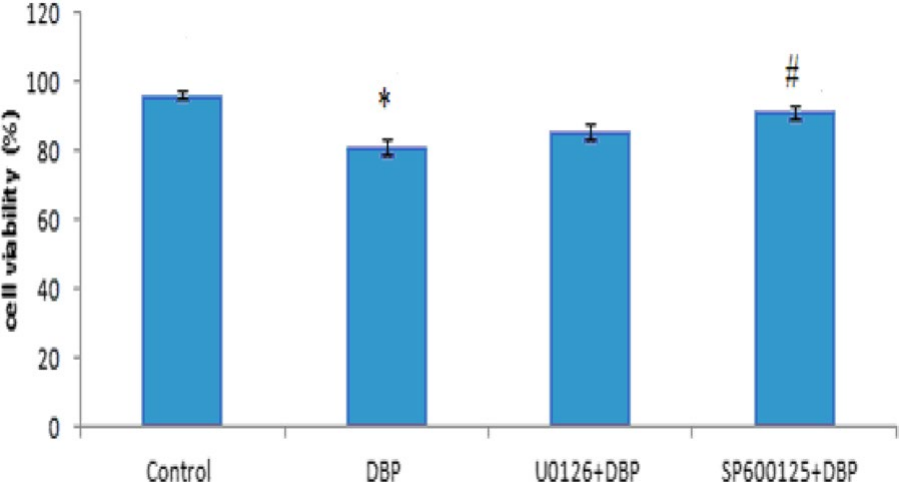
Cell proliferation rate of Sertoli cells induced by DBP in each group. *P < 0.05 vs. the control group; ^#^*P *< 0.05 vs. DBP groups; *n *= 8 in each group

The results of MTT showed that DBP could significantly reduce the proliferation of Sertoli cells (see Fig. 3), and the viability rate of the cells was 81.5 ± 2.32. Compared with the solvent control group (the cell survival rate was 96.4 ± 1.28), the difference was statistically significant (P < 0.05). The ERK1/2 inhibitor (U0126) and the JNK inhibitor (SP600125) were added to pre-treat the Sertoli cells 2 h, then added DBP, Sertoli cell viability rate increased. The viability rates of U0126 + DBP and SP600125 + DBP cells were 85.7 ± 2.12 and 91.4 ± 1.72, respectively. Compared with the DBP group, cell viability rate in JNK inhibitor (SP600125) + DBP group increased statistically significant.

### Effects of DBP on Sertoli cell apoptosis rate

**Fig. 4:**
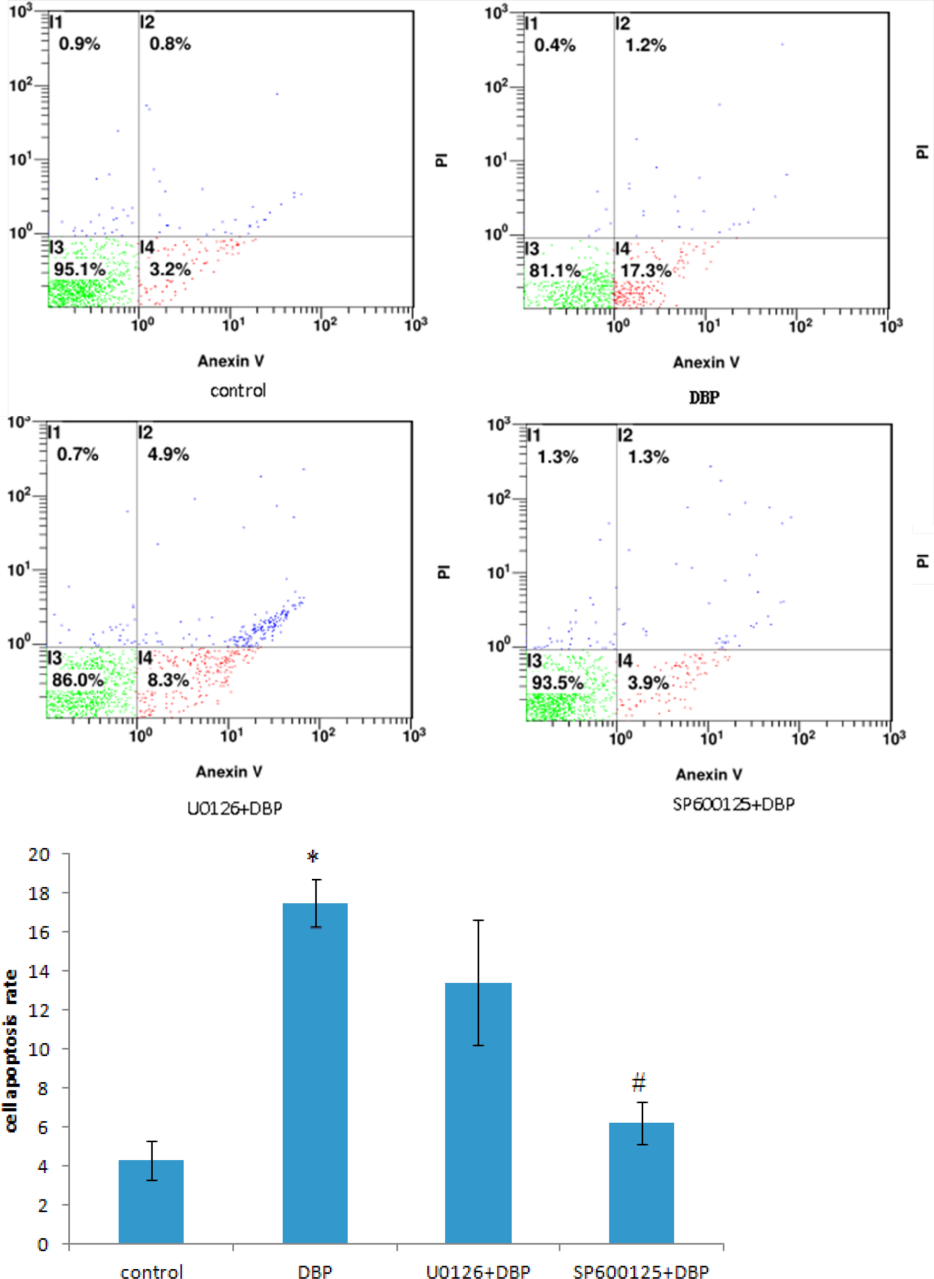
Sertoli cell apoptosis induced by DBP in each group. *P < 0.05 vs. the control group; ^#^*P *< 0.05 vs. DBP groups; *n *= 8 in each group

The results of flow cytometry (Fig. 4) showed that compared with the control group (the apoptotic rate of the cells was 4.32 ± 0.98), DBP significantly induced the apoptosis of the Sertoli cells, and the apoptotic rate was 17.5 ± 1.21. After the pretreatment of the cells with ERK1/2 inhibitor (U0126) and JNK inhibitor (SP600125), cell apoptosis rate decreased. The apoptosis rates of U0126 + DBP and SP600125 + DBP cells were 13.4 ± 3.21 and 6.23 ± 1.08, respectively. Compared with DBP group, the apoptotic rate decrease in JNK inhibitor (SP600125) + DBP group was statistically significant (P < 0.05).

### DBP induces activation of JNK and ERK1/2 signaling pathway in the Sertoli cells

**Fig. 5:**
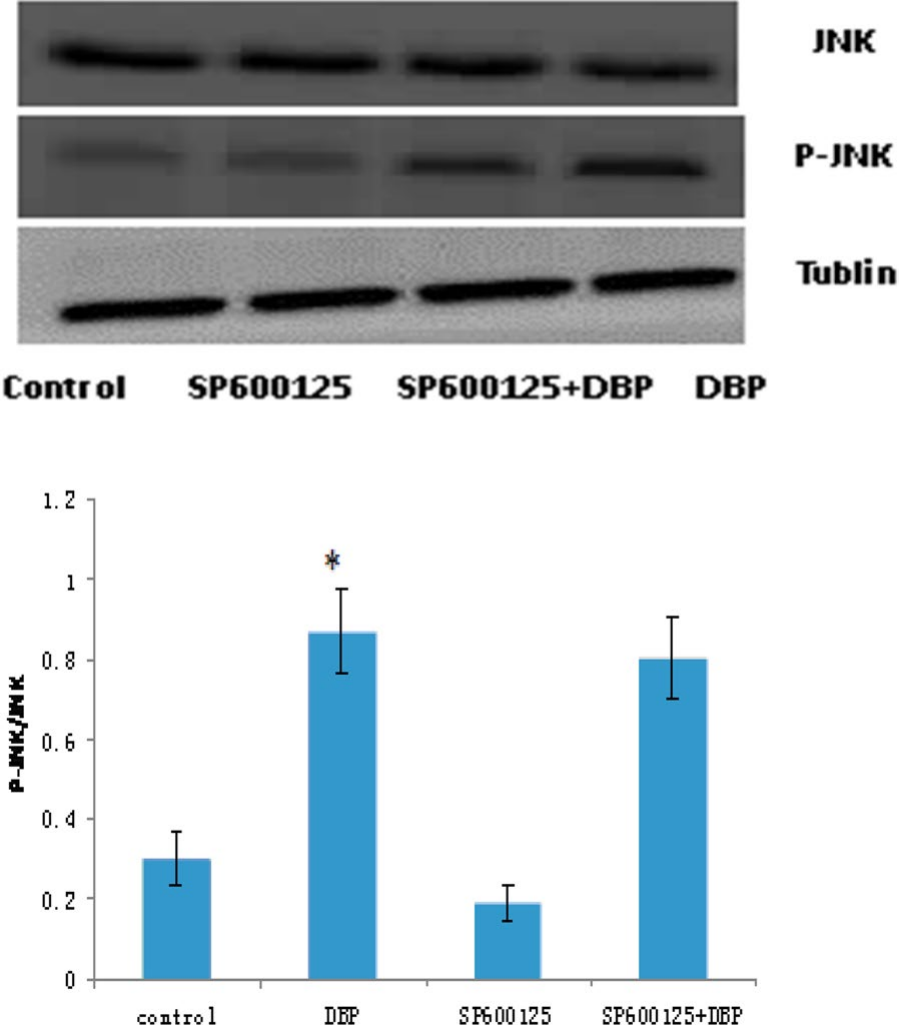
JNK and p-JNK expression levels in Sertoli cell. *P < 0.05 vs. the control group; *n *= 8 in each group

**Fig. 6:**
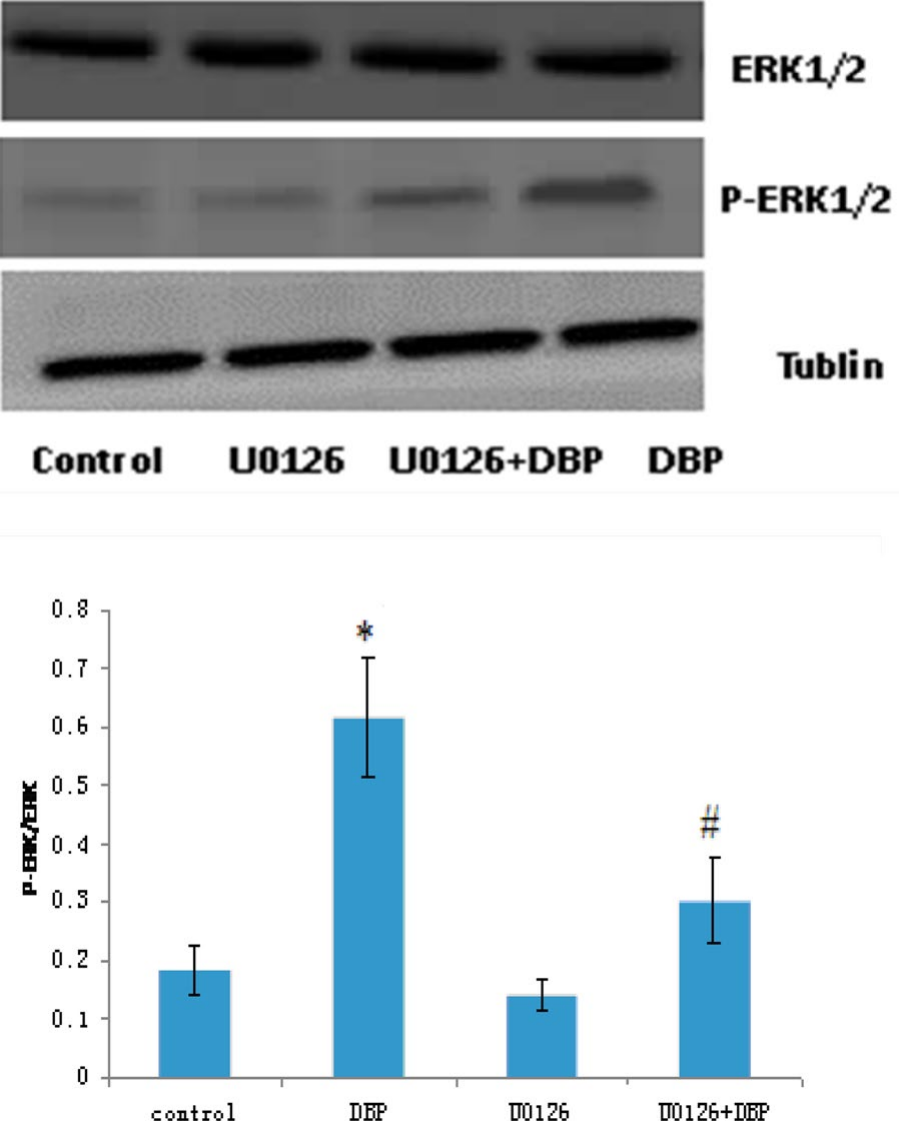
ERK1/2 and p-ERK1/2 expression levels in Sertoli cell. *P < 0.05 vs. the control group; ^#^*P *< 0.05 vs. DBP groups;. *n *= 8 in each group

Pretreatment with U0126 and SP600125, respectively to primary Sertoli cells for 2 h, then add 100 μg/mL DBP, Western blot (Figs. 5, 6) showed that compared with the DBP group alone, the phosphorylated JNK was not significantly decreased in the JNK inhibitor (SP600125) + DBP group and the phosphorylated ERK1/2 expression was significantly decreased in the ERK1/2 inhibitor (U0126) + DBP group. The ratio of the optical density of the phosphorylated protein to the corresponding total protein in Sertoli cell was seen in Table 4. Compared with the solvent control group, the ratio of p-ERK/ERK and p-JNK/JNK in DBP group increased, the difference was statistically significant (P < 0.05). ERK inhibitors (U0126) and JNK inhibitors (SP600125) reduced the expression of phosphorylated ERK and JNK. Compared with the DBP group, the ratio of p-JNK/JNK was not significantly decreased in the JNK inhibitor (SP600125) + DBP group, while the ratio of p-ERK/ERK was significantly decreased (P < 0.05). These findings indicated that DBP mainly activates the phosphorylation of JNK and participates in the cell damage.

**Table 4:** The ratio of the optical density of p-JNK and p-ERK to JNK and ERK in Sertoli cell

Group	P-ERK/ERK	P-JNK/JNK
Control	0.1838 ± 0.0431	0.3033 ± 0.0694
DBP	0.6167 ± 0.1018*	0.8691 ± 0.1042*
U0126	0.1414 ± 0.0274	–
U0126 + DBP	0.3027 ± 0.0725^#^	–
SP600125	–	0.1901 ± 0.0434
SP600125 + DBP	–	0.8055 ± 0.1046

*P < 0.05 vs. the control group; ^#^*P *< 0.05 vs. DBP groups; *n *= 8 in each group

## Discussion

As an environmental endocrine disruptor, DBP has certain male reproductive toxicity, can cause male rodents testicular atrophy, weight loss, decreased testicular activity of the enzyme, seminiferous tubules atrophy, spermatogenic cell loss, genital malformations, etc. [17]. In this vivo study of testicular pathology showed that DBP lead to testicular tissue structure damage, manifested as spermatogenic tubule disorders, Sertoli cell vacuolization, germ cell shedding, and so on. Sperm quality assessment can reflect the ultimate toxic effects of environmental factors on male reproductive damage. We found that DBP can reduce the sperm count and viability of male animals, increased sperm deformity rate, indicating that DBP interfere with the spermatogenesis process, showing male reproductive toxicity. But the dose response relationship is only shown in the sperm malformation rate, and in the other measured parameters the effect was only observed at high doses The reason for our analysis may be: changes in testicular tissue morphology after DBP exposure indicated that different doses of the dose DBP can penetrate the blood-testis barrier, enter the testicular cells, damage the Leydig cells and Sertoli cells, cause spermatogenesis disorders, interfere with sperm growth and development, and ultimately lead to increased sperm aberration rate. Sperm activity reflects the quality of active sperm. The highest dose of DBP in this paper can significantly reduce sperm motility. It may be that at this high dose DBP inhibited certain enzymes in sperm energy metabolism. Impaired sperm energy metabolism, resulting in sperm stagnation, or sperm damage and increased sperm deformity rate leading to impaired exercise capacity. Male reproductive function is mainly controlled by hypothalamic–pituitary–testicular axis. Hypothalamic secrete gonadotropin-releasing hormone (GnRH), GnRH act on the pituitary gland, the latter secrete FSH, LH, which act respectively on the testicular spermatogenic cells, Sertoli cells and Leydig cells, cause testosterone androgen secretion, achieve the start and regulation of spermatogenesis. Detection of serum reproductive hormones can determine the state of testicular damage. The results of this study show that compared with the control group, rat serum FSH, LH of high-dose DBP group was significantly increased and testosterone levels decreased significantly. The results of reproductive toxicity of environmental endocrine disruptors DBP in male rats in many literatures are generally reduced testosterone secretion, while the results reported on FSH and LH are different [18–20], we analyzed that the difference results is related to DBP dose, the time of exposure, the type, age of animal and other factors. But these suggested that DBP indirect damage to the testis through endocrine disruption, but also that Sertoli cells may be the direct target of DBP toxic effects [21]. Sertoli cells present in the testicular spermatogenic epithelium, the structure and function integrity of Sertoli cells is critical to the proliferation and maturation of spermatogenic cells. In this study, we used MTT assay to detect the inhibitory effect of DBP on the proliferation of Sertoli cells. It was found that DBP significantly inhibited cell proliferation compared with the control group. Flow cytometry showed that DBP-treated cell apoptotic rate was significantly higher than that the control group. The results confirm that DBP induces cytotoxicity in Sertoli cells causing apoptosis in Sertoli cells cultured in vitro.

Many studies have found that male reproductive health have a relationship between MAPKs pathway. MAPKs involved in spermatogenesis [22], germ cell development and maturation [23], germ cell apoptosis [24]. We examined the phosphorylation of ERK1/2, JNK, p38 by Western blot to confirm whether DBP will interfere with MAPKs signaling pathway. In the *vivo* experiment study, the ratio of p-ERK/ERK and p-JNK/JNK in MAPKs-related proteins increased compared with the solvent control group, the difference was statistically significant (P < 0.05), while the changes of p-P38/P38 were not significant, indicating that JNK, ERK phosphorylation level was significantly increased, and P38 phosphorylation level was not significant. These suggests that the JNK and ERK pathways in the MAPKs signal are involved in testicular damage. This result is not completely consistent with the relevant research reports. Qi et al. believe that JNKs/p38 MPAK is involved in the apoptosis of Sertoli cells [25]. Song et al. believe that p38 MPAK mainly plays a major role in Sertoli cell injury [26], Choi et al. believe that ERK pathway plays a major role in Sertoli cell damage [27]. This difference is due to the different reproductive chemical toxicants studied by the researchers, and different animal species and cell lines used in vivo and in vitro studies.

In addition, in order to further confirm the mechanism of JNK and ERK-MAPKs signaling pathway on testicular injury, we used testicular primary Sertoli in vitro experiments. The results showed that compared with the solvent control group, DBP could increase the phosphorylation of MAPKs-related proteins ERK and JNK, and the ratio of p-ERK/ERK and p-JNK/ERK increased, the difference was statistically significant (P < 0.05), indicating that DBP can activate the JNK and ERK-MAPKs signaling pathway of testicular Sertoli cells. To further confirm whether the phosphorylation of JNK and ERK1/2 is involved in apoptosis and whether the two effects are equal or which dominates, we used ERK1/2 inhibitors (U0126) and JNK inhibitors (SP600125) pretreatment primary Sertoli cells 2 h, and then incubated with 100 μg/mL of DBP. The results showed that ERK1/2 inhibitors (U0126) and JNK inhibitors (SP600125) reduced the expression of phosphorylated ERK1/2 and JNK, compared with the DBP group, the apoptosis rate of U0126 + DBP group and SP600125 + DBP group decreased, the cell proliferation rate increased, the ratio of p-ERK/ERK and p-JNK/ERK decreased, indicating that the inhibitors of JNK and ERK were all can weaken DBP-induced cell apoptosis, increase cell survival, reduce ERK1/2 and JNK phosphorylation. Furthermore, it was found that cell viability rate increase and the apoptotic rate decrease in JNK inhibitor (SP600125) + DBP group was more obvious, the difference was statistically significant (P < 0.05), indicating that the repress role of apoptosis by JNK inhibitors is more prominent. Western blot showed that compared with DBP group, the ratio of p-JNK/ERK was not significantly decreased in JNK inhibitor (SP600125) + DBP group, but in ERK1/2 inhibitor (U0126) + DBP group, the ratio of p-ERK/ERK was significantly decreased. These findings illustrated that SP600125, a selective inhibitor of JNK, was shown to repress the DBP-induced JNK phosphorylation/activation in Sertoli cells, which indicated that DBP mainly activates JNK phosphorylation. So  collectively, in vivo and in vitro study results, indicate that DBP mediates its disruptive effects at the Sertoli cell via the JNK-MAPK signaling pathway. These findings provide insightful information regarding a therapeutic approach to DBP-induced male infertility, such as via the use of specific JNK-MAPK inhibitor.

In summary, the results of this experiment indicate that the activation of MAPKs signaling pathway, especially JNK, may participate in the damage of DBP to testicular Sertoli cells. In addition, our previous in vitro results showed that the PTEN/PI3 K/AKT/mTOR signaling pathway plays an important role in DBP-induced apoptosis of testicular Sertoli cells [28]. These suggested that MAPK and Akt pathways both can mediate support cell death caused by reproductive toxicants which coincides with that nonylphenol-induced apoptosis in mouse Sertoli cell line TM4 cells can be mediated by MAPK and Akt pathways [29]. As noted herein, multiple pathways such as both Akt and MAPK signal pathways are involved in chemical toxicant-induced testicular injury, and research is needed to delineate the molecular mechanism(s) that regulates crosstalk between these signal pathways, which will be helpful in gaining insightful information to intervene toxicant induced testicular injury.

## Conclusions

In conclusion, DBP can lead to testicular toxicity: decreased testosterone in blood serum, leaded to sperm reduction and malformation and even damaged the normal structure of seminiferous tubules. The activation of MAPKs signaling pathway, especially JNK, may participate in the damage of DBP to testicular Sertoli cells. We need to study more deeply such as the molecular mechanism(s) about signal pathways, which will be helpful in gaining insightful information to intervene the reproductive toxicity of DBP.

## Methods

### Animals and treatments

Male clean Sprague–Dawley rats (4 weeks old) were obtained from the Laboratory Animal Center of Jilin University (Jilin, China). All experimental protocols were conducted in accordance with the principles and procedures outlined in the “Guide for Care and Use of Laboratory animals” and approved by the Ethics Committee for the Use of Experimental Animals of Beihua University.

Rats were housed under a 12:12 h light–dark cycle with well ventilation and constant temperature (26 ± 1 °C). Animals were adapted to laboratory lighting and feeding condition for 1 week before experiment. Rats were allowed free access to food and drinking water. After adaptation, male SD rats were assigned randomly to four group (n = 8) and administered corn oil (vehicle control) or DBP (analytical grade, purity 99.5%) (Sigma, USA) at doses of 50, 500 and 1000 mg/kg/day by gavage for 35 days. Animals were sacrificed by decapitation after the last treatment and the blood, testes and epididymides were isolated immediately for the following analyses.

### Measurement of reproductive hormone

The blood samples were centrifuged at 2000 rpm, 4 °C for 10 min and testosterone (T), follicle-stimulating hormone (FSH) and luteinizing hormone (LH) in serum were detected using ELISA kits according to the manufacturer’s instructions (Shanghai Jiang Lai biological company, China).

### Sperm analysis

The fresh epididymides were weighted then cut longitudinally and put in 2 mL 0.9% sodium chloride solution for 10 min at 35 °C to release sperms into the media. The suspension was filtered using nylon mesh and adjusted to an adequate concentration. Sperm count, viability rate and malformation rate were determined using WLJY-9000 color sperm quality detection system (Beijing Weili Inc., China).

### Testis histological examination

Hematoxylin–eosin (HE) staining was applied to testes. Tissues were immersed in Bouin’s solution (picric acid-aqueous solution: formaldehyde:glacial acetic acid, 15:5:1) for 24 h at room temperature, embedded in paraffin wax and sectioned into 5 μm thick slices. After dewaxed in xylol, dehydrated in ethanol series and washed in water, slices were stained with hematoxylin and eosin, following the standard HE staining procedures. Images were taken by a digital camera (DP20, Olympus, Tokyo, Japan) to show the extent of testes injury.

### Isolation of Sertoli cells

Primary Sertoli cells were isolated from 18-day-old male rats. Briefly, animals were euthanized by CO_2_ asphyxiation, and testes were isolated, decapsulated, and cut into small pieces. After washed twice in DMEM-F12 (Hyclone, USA), fragments were digested with 0.1% (w/v) trypsin (Difco, USA) for 30 min then centrifuged twice at 800 rpm for 2 min. Cell debris was resuspended in 0.05% (w/v) collagenase I (Sigma, USA) with gentle pipetting using Pasteur pipette and digested for about 10 min, until seminiferous tubules were nearly invisible. Suspension was filtered through nylon mesh and centrifuged for 5 times. Resuspend cells with DMEM-F12 containing 15% fetal bovine serum (Hangzhou Sijiqing, China) and plate cells on 100 mm dishes at a density of 0.5 × 10^5^ cells/cm^2^. High purity Sertoli cells were obtained after incubated for 36 h at 35 °C in 5% CO_2_ followed by treated with 20 mM Tris–HCl buffer (pH 7.4) for 2.5 min to remove residual germ cells. The purity of Sertoli cells was routinely more than 90% [30]. On the 4th day of in vitro culture, Sertoli cells were ready for the subsequent treatments.

### Treatment of Sertoli cells

The cells were divided into solvent control group and experimental group. Solvent control group was adding 0.1% Dimethylmain (DMSO, Amresco, USA) in cells, the experimental group were differently DBP group (DBP dose: 100 μg/mL), ERK1/2 inhibitor (U0126) (Calbiochem, USA) + DBP group (U0126 dose: 10 μmol/mL + DBP dose: 100 μg/mL) and JNK inhibitor (SP600125) (Calbiochem, USA) + DBP group (SP600125 dose: 10 μmol/mL + DBP dose: 100 μg/mL). ERK1/2 inhibitors (U0126) and JNK inhibitors (SP600125) pretreatment primary Sertoli cells 2 h, and then incubated with 100 μg/mL of DBP 24 h.

### MTT assay

Sertoli cells were transferred into a 96-well plate at a density of 1 × 10^5^ cells/mL. Cell viability was estimated by MTT assay after 24 h treatment. Cells were incubated in serum-free media containing 20 μL MTT (5 mg/mL) for 4 h. 150 μL DMSO was added after discarding culture media. Shake for 10 min, and the absorbance were acquired at 490 nm by microplate reader (Tecan, Switzerland).

### Sertoli apoptosis rate detection

After treatment for 24 h, the cells were digested and trypsinized, then harvested. The cells were treated according to the Annexin v-FITC/PI kit instructions (Biovision, USA). The cells were mixed with 400 μL binding buffer and then 4 μL PI and 4 μL Annesxin v were added. The cells were incubated at room temperature for 15–20 min at in dark room. FACS420 flow cytometer (Becton–Dickinson, USA) was used to detect cell apoptosis.

### Western blot analysis

In vivo experiments, testicular tissue of each group rats was removed and quickly placed in liquid nitrogen to be stored for inspection. The sample was placed in 4 °C pre-cooling lysis buffer, 4 °C ultrasonic mixing (6 s × 6 times), 4 °C incubated for 30 min, centrifuged 25,000 rpm, 10 min × 3 times, the supernatant sub-assembly; The content of protein was determined by Bradford method, and the protein was separated by 10% SDS-PAGE using standard bovine serum albumin. Each well was loaded with 60 μg of protein and electrophoresis was performed at a constant voltage of 160 V until the bottom of the gel was transplanted. After blocked with 5% non-fat dry milk for 2 h, 1: 100 rabbit anti-rat ERK1/2, p-ERK1/2, JNK, p-JNK, P38, p-P38 and tubulin monoclonal antibody (Beijing Solarbio, China) were added, shaken at 37 °C for 1 h, Washed three times with phosphate buffer solution-Tween 20 for 10 min. The conjugate horseradish peroxidase-labeled secondary antibody (1: 300) was added, shaken at 37 °C for 1 h, washed in PBST for 3 times and irradiated with chemiluminescence agent.

In vitro experiments, Sertoli cells were homogenized in lysis buffer on ice for 30 min and the homogenate was collected followed by centrifugation at 1000 rpm for 10 min at 4 °C. The protein concentration of supernatant was measured. Equal amounts of proteins (40 μg) were loaded on 10% polyacrylamide gel with 4% stacking gel to apply SDS-PAGE and then transferred to nitrocellulose membranes. After blocked with 5% nonfat milk for 1 h at room temperature, the membranes were incubated with primary antibodies (JNK, p-JNK, ERK1/2, p-ERK1/2, and tubulin, 1:1000 dilution) overnight at 4 °C. Protein blots were revealed by horseradish peroxidase-conjugated secondary antibody (1:3000 dilution) and visualized by enhanced chemiluminescent kit.

Proteins were quantified by densitometry with the ImageJ software (National Institutes of Health, USA). Data were normalized against tubulin in each group.

### Statistical analysis

Data in this study were presented as mean ± SD and determined by one-way analysis of variance (ANOVA) followed by Bonferroni’s test. Difference was considered statistically significant at *P *< 0.05.

## Data Availability

The data are available from the corresponding author.

## Abbreviations

DBP: di-*N*-butyl-phthalate
MAPK: mitogen-activated protein kinase
JNK: c-Jun N-terminal kinase
ERK: extracellular signal regulated kinase
FSH: follicle stimulating hormone
T: testosterone
LH: luteinizing hormone
